# Roflumilast Inhibits Lipopolysaccharide-Induced Tumor Necrosis Factor-α and Chemokine Production by Human Lung Parenchyma

**DOI:** 10.1371/journal.pone.0074640

**Published:** 2013-09-16

**Authors:** Amparo Buenestado, Marie-Camille Chaumais, Stanislas Grassin-Delyle, Paul-André Risse, Emmanuel Naline, Elisabeth Longchampt, Hermann Tenor, Philippe Devillier

**Affiliations:** 1 Laboratory of Pulmonary Pharmacology UPRES EA220, University of Versailles Saint-Quentin en Yvelines, Suresnes, France; 2 Department of Pathology, Foch Hospital, Suresnes, France; 3 Nycomed GmbH: A Takeda Company, Konstanz, Germany; University of Giessen Lung Center, Germany

## Abstract

**Background:**

Roflumilast is the first phosphodiesterase-4 (PDE4) inhibitor to have been approved for the treatment of COPD. The anti-inflammatory profile of PDE4 inhibitors has not yet been explored in human lung tissues. We investigated the effects of roflumilast and its active metabolite roflumilast-N-oxide on the lipopolysaccharide (LPS)-induced release of tumor necrosis factor-alpha (TNF-α) and chemokines by human lung parenchymal explants. We also investigated roflumilast’s interaction with the long-acting β_2_-agonist formoterol.

**Methods:**

Explants from 25 patients undergoing surgical lung resection were incubated with Roflumilast, Roflumilast-N-oxide and formoterol and stimulated with LPS. Levels of TNF-α, chemokines (in the culture supernatants) and cyclic adenosine monophosphate (in tissue homogenates) were determined with appropriate immunoassays.

**Results:**

Roflumilast and Roflumilast-N-oxide concentration-dependently reduced the release of TNF-α and chemokines CCL2, CCL3, CCL4, CXCL9 and CXCL10 from LPS-stimulated human lung explants, whereas CXCL1, CXCL5 and CXCL8 release was not altered. Formoterol (10 nM) partially decreased the release of the same cytokines and significantly increased the inhibitory effect of roflumilast on the release of the cytokines.

**Conclusions:**

In human lung parenchymal explants, roflumilast and roflumilast-N-oxide reduced the LPS-induced release of TNF-α and chemokines involved in the recruitment of monocytes and T-cells but not those involved in the recruitment of neutrophils. Addition of formoterol to roflumilast provided superior *in* *vitro* anti-inflammatory activity, which may translate into greater efficacy in COPD.

## Introduction

Much of the pharmacological interest in the phosphodiesterase (PDE)-4 family of enzymes relates to their broad, functional role in inflammatory, immunocompetent and structural cells involved in respiratory disorders such as chronic obstructive pulmonary disease (COPD) [1]. Roflumilast is the first PDE4 inhibitor to be marketed and has been approved for oral, once-daily treatment of severe COPD associated with chronic bronchitis in patients with frequent exacerbations (along with bronchodilator treatment) [2]. Indeed, roflumilast’s most interesting clinical feature is probably its ability to reduce exacerbation rates in the frequent-exacerbation phenotype of COPD [3,4]. In the liver, roflumilast is converted to roflumilast-N-oxide, an active metabolite with much the same potency and selectivity for PDE4 as the parent compound [1,5]. It has been estimated that roflumilast-N-oxide accounts for about 90% of the overall, roflumilast-related PDE4 inhibition *in vivo* [6]. After repeated oral administration of roflumilast at the clinically recommended dose (500 µg/d), plasma levels of free (unbound) roflumilast-N-oxide remained at about 1-2 nM over the 24h dosing interval [7]. In this concentration range, both roflumilast and roflumilast-N-oxide favorably modulate *in vitro* inflammatory responses in human cells involved in COPD [1,5,8].

The potential effects of roflumilast and roflumilast N-oxide on the LPS-induced release of cytokines and chemokines from the complex cellular network in human lung parenchyma have not previously been explored. Lipopolysaccharide (LPS) is a major component in the cell walls of Gram-negative bacteria, which are thought to cause a substantial portion of COPD exacerbations [9-11].

The aim of the present study in human lung explants (in which the *in situ* parenchymal architecture and cell-cell communications are maintained [12-14]) was to determine whether roflumilast and/or roflumilast-N-oxide reduce the LPS-induced release of cytokines and particularly tumor necrosis factor (TNF)-α and chemokines involved in the recruitment of T-lymphocytes (CCL3, CCL4, CXCL9 and CXCL10), monocytes (CCL2) and neutrophils (CXCL1, CXCL5 and CXCL8) [15-18]. Infiltration of the lungs by these inflammatory cell types is a hallmark of COPD [19].

Long-acting bronchodilators are among the pillars of COPD treatment. Long-acting β_2_-agonists (LABAs) such as formoterol relax airway smooth muscle by increasing the intracellular concentration of cyclic adenosine monophosphate (cAMP). β_2_-adrenoceptors are expressed on most structural and inflammatory cells in the lungs. It has been shown that activation of these receptors enhances the anti-inflammatory effects of glucocorticoids [20]. In both asthma and (to a lesser extent) COPD, inhaled combinations of LABAs and glucocorticoids prevent acute exacerbations more effectively than the components alone [20-22]. Since roflumilast and roflumilast-N-oxide prevent the breakdown of cAMP by PDE4 family enzymes, *in vitro* β_2_-adrenoceptor agonists may further increase intracellular cAMP levels and thereby enhance the PDE4 inhibitors’ anti-inflammatory effects. Therefore, the present study’s second objective was to assess the effects of formoterol (alone and combined with roflumilast) on human lung explants in terms of (i) LPS-induced release of TNF-α or chemokines and (ii) cAMP formation.

## Methods

### Reagents

Penicillin-streptomycin, L-glutamine, cAMP, ethylene glycol tetraacetic acid (EGTA) and erythro-9-(2-hydroxy-3-nonyl) adenine hydrochloride (EHNA), 8-methoxymethyl-IBMX, cilostamide, β-mercaptoethanol, glycerol, aprotinin, leupeptin, pefabloc, benzamidine, soybean trypsin inhibitor, RPMI, formoterol fumarate dihydrate, forskolin, rolipram, fetal calf serum, indomethacin, DMSO and LPS (from *E. coli* serotype 0111:B4) were purchased from Sigma (St. Louis, MO). Diethylether and trichloroacetic acid were from VWR Prolabo (Fontenay-sous-Bois, France). Triton was supplied by Eurobio Biotechnology (Les Ulis, France). [^3^H]-cAMP was from Amersham (Aylesbury, UK). Roflumilast and roflumilast N-oxide were synthesized at Nycomed GmbH-Takeda (Konstanz, Germany). All other chemicals were of analytical grade and were obtained from Prolabo (Briare, France).

### Patient population

The use of resected lung tissues was approved by the regional investigational review board (*Comité de Protection des Personnes Ile de France VIII*, Boulogne-Billancourt, France; accession numbers: DC 08 09 45 and DC 11 10 05) and written informed consent was obtained from each patient. Lung tissues were obtained from 25 patients (mean±SD age: 63±3 years; M/F=18:7; FEV _1_/FVC ratio: 0.86±0.06; smokers/ex-smokers: 14:11, pack-years: 41±5) undergoing surgical lung resection. None of the patients had undergone prior chemotherapy or radiotherapy.

### Preparation of human lung parenchyma explants

**Figure 1:**
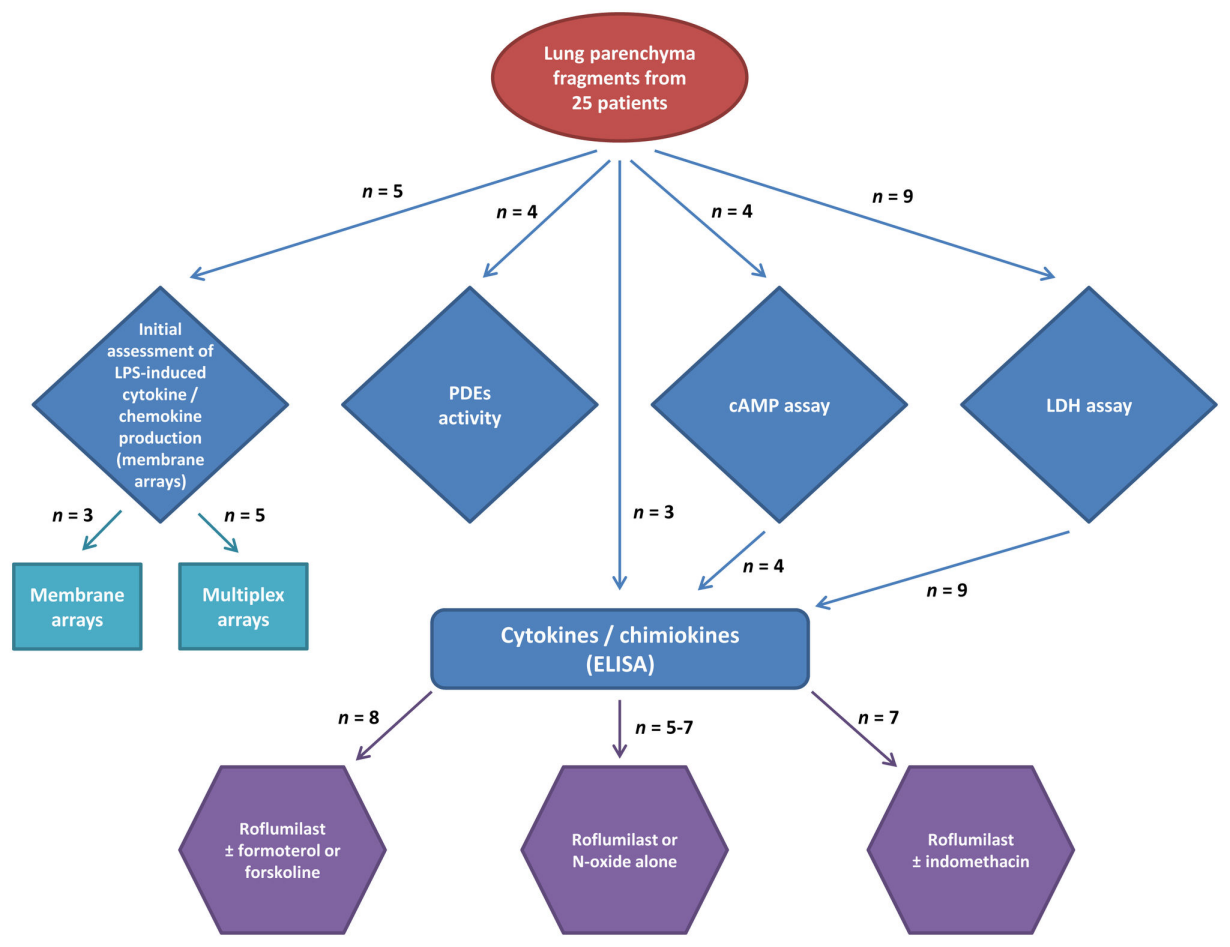
Flow-chart of human samples used in the different series of experiments.

The procedure for preparation of lung explants has been described elsewhere [13,14]. Briefly, all tissue specimens were taken from areas lacking macroscopic signs of infection, necrosis or ischemia and at some distance from the tumour. The specimens were dissected free of pleura, visible airways and blood vessels and were finely chopped into 3-5 mm^3^ fragments. After several washes in RPMI supplemented with 2 mM L-glutamine, 100 µg/mL streptomycin and 100 U/mL penicillin (to prevent contamination by blood), the explants were maintained overnight at 4°C. The fragments were distributed into 6-well plates (5 fragments and 5 mL of supplemented RPMI per well, total weight~50-100 mg) and incubated with roflumilast or roflumilast-N-oxide (0.1-1000 nM) or vehicle (DMSO) for 1h prior to LPS stimulation (1 µg/mL). After 24h, supernatants were collected and stored at -80 °C for subsequent cytokine assays. The optimal incubation time (24h) and LPS concentration (1 µg/mL) had been determined previously. We checked for the absence of endotoxin in controls (E-toxate kit, Sigma). In some experiments, formoterol (10 nM) or forskolin (10 µM) were added together with roflumilast or vehicle. Stock solutions were prepared in DMSO. The maximum DMSO concentration in culture wells (0.2%) did not affect LPS-induced cytokine release. All wells were run in duplicate. Details of tissue samples used in each series of experiments are shown in Figure 1.

### Assay of PDE activities in parenchymal explants

Following exposure to LPS (1 µg/mL) or medium for 4h, lung explants (100 mg) were homogenized in 1 mL of buffer (100 mM Tris-HCL pH 7.4, 2 mM MgSO_4_, 2 mM EDTA, 10% glycerol, 1 mM β-mercaptoethanol, 10 µg/mL aprotinin, 1 µM leupeptin, 25 µg/mL pefabloc, 130 µg/mL benzamidine and 50 µg/mL soybean trypsin inhibitor) with an Ultra-Turrax homogenizer (IKA, Staufen, Germany) and cells were disrupted by sonication for 3×15s in a Branson sonifier (Branson Ultrasonics, Geneva, Switzerland). Lysates were immediately tested for PDE activity in a radioenzymatic assay, as described previously [23]. [^3^H]-3’,5’-cAMP (20.000 c.p.m.) was used as a tracer and the total cAMP hydrolyzing activity and PDE3 and PDE4 activities were measured at a cAMP substrate concentration of 1 µM in the presence of 10 µM cilostamide (to block PDE3) and 10 µM rolipram (to block PDE4). The protein concentration was determined (in a Bio-Rad protein assay) in order to calculate the specific activity (picomoles cAMP hydrolyzed per minute per mg of protein).

### Cytokine release profiling with cytokine arrays

**Figure 2:**
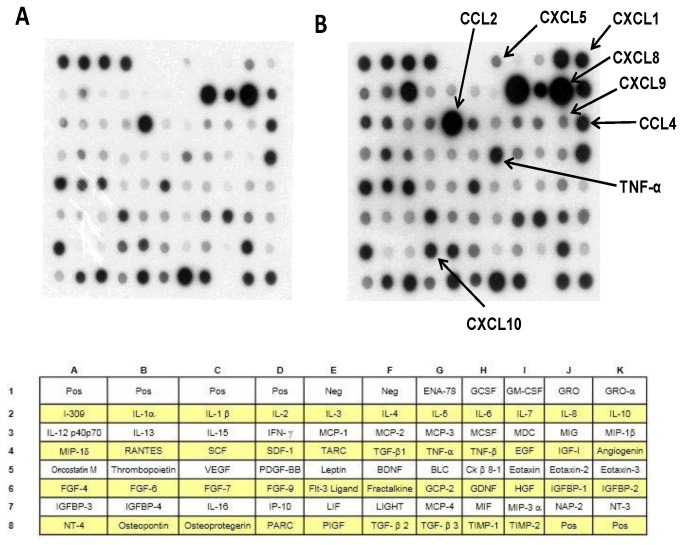
Cytokine antibody array membranes. Parenchymal explants were incubated with medium (A) or LPS (1 µg/mL) (B) for 24h. 1 mL of supernatant was used with each array membrane. The array data are representative of three independent experiments.

The secretion of cytokines/chemokines by human lung parenchymal explants was evaluated using a protein array method (RayBio^®^ Human Cytokine Antibody Array V, RayBiotech Inc., Le Perray en Yvelines, France). The assay is capable of simultaneously detecting 73 different cytokines (including monokines, interleukins, chemokines, and growth factors (Figure 2)) with high specificity. The sensitivity of the antibodies present in the array ranged from 1 to 2000 pg/mL. The relative concentrations of the cytokines in the media were determined from the densities of individual spots measured with QuantityOne 4.2.1 (Bio-Rad, Marnes-La-Coquette, France).

Cytokine secretion was also assessed with two SearchLight Multiplex Arrays (Pierce Biotechnology Inc., Rockford, IL). The human TH _1_/TH_2_ array 1 detects 9 cytokines (TNF-α, IL-2, IL-4, IL-5, CXCL8, IL-10, IL-12p70, IL-13, IFN-γ). The custom human 16-plex array detects 15 cytokines (IL-1α, IL-1β, IL-7, IL-9, GM-CSF, CCL1, CCL2, CCL4, CCL5, CXCL1, CXCL5, CXCL7, CXCL12, fibroblast growth factor (FGF)-β, vascular endothelial growth factor (VEGF)) and matrix metalloproteinase 9. The arrays’ limits of detection (LODs) ranged from 0.2 to 20 pg/mL. The results of these two arrays were read with a cooled charge-coupled device (CCD) camera and the Searchlight CCD imaging and analysis system. All the cytokine measurements were performed on conditioned media obtained after the incubation of lung explants in the absence or presence of LPS (1 µg/mL) for 24h at 37°C and with 5% CO_2_.

### Immunoassays and enzyme assays

Cytotoxicity was assessed by measuring lactate dehydrogenase (LDH) levels in supernatants after 24h of culture using an enzyme assay (Cayman Chemical, Tallinn, Estonia). The detection limit was 300 µU/mL. Parenchymal explants homogenized in Triton X100 were used as positive controls.

Cytokines were measured by ELISA (Duoset Development System, R&D Systems, Lille, France) and the results were expressed as ng/100 mg of wet tissue. The LODs were 4 pg/mL for CCL3, 8 pg/mL for TNF-α, CCL2, CCL4 and CXCL5, 16 pg/mL for CXCL1, CXCL8 and CXCL10 and 32 pg/mL for CXCL9.

Levels of PGE_2_ in lung explant supernatants and cAMP in lung parenchymal homogenates were assessed using commercially available enzymo-immunoassay kits (Cayman Chemical) with LODs of 36 pg/mL and 3.1 pmol/mL respectively. For cAMP, lung parenchymal explants were weighed and incubated for either 20 min or 2h with LPS (1 µg/mL), roflumilast (1 or 100 nM), or formoterol (10 nM) or vehicle (0.1% DMSO), as indicated. Tissues were immediately frozen in liquid nitrogen and extracted for the cAMP assay [12]. Briefly, frozen tissues were homogenized in 50 mM phosphate buffer and 5% trichloroacetic acid solution using a motor-driven homogenizer. The homogenates were centrifuged at 1500 g for 10 min at 4°C and the supernatant was transferred into another tube. The trichloroacetic acid was extracted with water-saturated diethylether and the residual ether was removed by heating the samples at 70°C for 5 min. Homogenates were harvested at -80°C.

### Statistical analysis

Data are expressed as means ± S.E.M.; *n* represents the number of patients from whom lung explants were obtained. Concentration-effect curves were plotted and analyzed using GraphPad Prism® software (GraphPad Software Inc., San Diego, CA). The potency (*pD*_*2*_) was defined as the negative log_10_ of the concentration producing 50% of the maximum inhibition (EC_50_). Data were compared in a one-way or repeated-measures analysis of variance, followed by Bonferroni’s post-hoc test. Statistical significance was defined as *p*<0.05.

## Results

### Lung explant viability

Under control conditions, the basal LDH level after 24h in the culture supernatants was 8.8 ± 1.7 mU/100 mg (n = 9). Exposure to LPS (1 µg/mL) did not cause significant cell injury, as indicated by the non-significant change in LDH release (9.6 ± 2.0 mU/100 mg; n = 9), which corresponded to less than 4% of the value observed for positive controls (259 ± 11 mU/100 mg, n = 9). Neither the vehicle nor any of the compounds used in this study enhanced LDH release.

### PDE enzyme activities in lung parenchymal explants

Total cAMP-PDE hydrolytic activity and PDE3 and PDE4 activities were measured in cell extracts of lung tissues. The overall cAMP-PDE activity at a substrate concentration of 1 µM amounted to 44.0 ± 4.3 pmol/min/mg of protein. In the presence of 10 µM cilostamide or 10 µM rolipram, the cAMP-PDE activities were 31.9 ± 2.1 pmol/min/mg of protein and 27.2 ± 2.8 pmol/min/mg of protein (n = 4), respectively. Based on these measurements, 27% and 38% of the cAMP hydrolyzing PDE activity corresponded to PDE3 and PDE4, respectively, at a cAMP substrate concentration of 1 µM. Exposure of lung parenchymal explants to LPS (1 µg/mL) for 4h affected neither the total cAMP-PDE activity (48.6 ± 6.2 pmol/min/mg of protein) nor the PDE3 and PDE4 activities (29.5 ± 3.2 pmol/min/mg of protein and 30.1 ± 3.6 pmol/min/mg of protein (n = 4), respectively).

### LPS-induced release of cytokines by human lung parenchymal explants

Screening (Figure 2) of supernatants from three independent experiments with protein arrays suggested a LPS-induced increase in the production of ENA-78 (CXCL5), GM-CSF, GRO (CXCL1, CXCL2, CXCL3), GRO-α (CXCL1), I-309 (CCL1), IL-1β, IL-6, IL-8, Il-10, IL-12 (p40/p70), IL-13, MCP-1 (CCL2), MIG (CXCL9), MIP-1β (CCL4), TNF-α, thrombopoietin, VEGF, FGF-6, FGF-7, Flt-3 ligand, IP-10 (CXCL10), and NT-4. Cytokine production was also assessed with SearchLight Multiplex Arrays (Table 1). From these screening steps, we selected 9 cytokines involved in COPD [15-19] for further experiments: TNF-α and chemokines involved in the recruitment of T-lymphocytes (CCL3, CCL4, CXCL9 and CXCL10), monocytes (CCL2) and neutrophils (CXCL1, CXCL5 and CXCL8) [15-18].

**Table 1:** LPS-induced cytokine release in culture supernatants of human lung explants.

Cytokine	No LPS	LPS	Fold change
TNF-α	25 ± 4	3209 ± 597	130
IL-10	19 ± 4	868 ± 161	46
IL-9	3975 ± 1122	117028 ± 26300	29
IL-1β	208 ± 42	4678 ± 1055	22
CXCL1	2053 ± 809	38910 ± 11687	19
CCL2	10236 ± 2452	175375 ± 34110	17
IL-1α	262 ± 65	3826 ± 1274	15
CCL4	6826 ± 1798	95602 ± 14809	14
CXCL8	32686 ± 9124	436755 ± 76061	13
GM-CSF	45968 ± 7960	575998 ± 150282	13
IL-7	614 ± 218	5985 ± 434	10
CCL5	282 ± 71	1805 ± 338	6
CXCL12	12362 ± 4128	71739 ± 15892	6
IL-6	92845 ± 20848	524726 ± 81444	6
IL-4	21 ± 4	107 ± 10	5
CXCL7	18562 ± 3384	85206 ± 11104	5
IL-5	10 ± 1	42 ± 5	4
CXCL5	3174 ± 485	9279 ± 2041	3
CCL1	639 ± 117	2136 ± 542	3
FGF-β	2895 ± 656	8865 ± 2090	3
VEGF	7574 ± 1808	24489 ± 6886	3
IL-13	623 ± 95	1008 ± 160	1.6

Cytokine levels (means ± S.E.M. pg/100 mg of lung tissue) were assessed with SearchLight Cytokine Multiplex Arrays after incubation in the absence or presence of LPS (1 µg/mL) for 24h (n = 5).

The concentrations of IL-12p70 and IFN-γ were close to or below the detection limits.

### Effects of roflumilast and its active metabolite roflumilast-N-oxide on TNF-α release from LPS-stimulated human lung parenchymal explants

**Figure 3:**
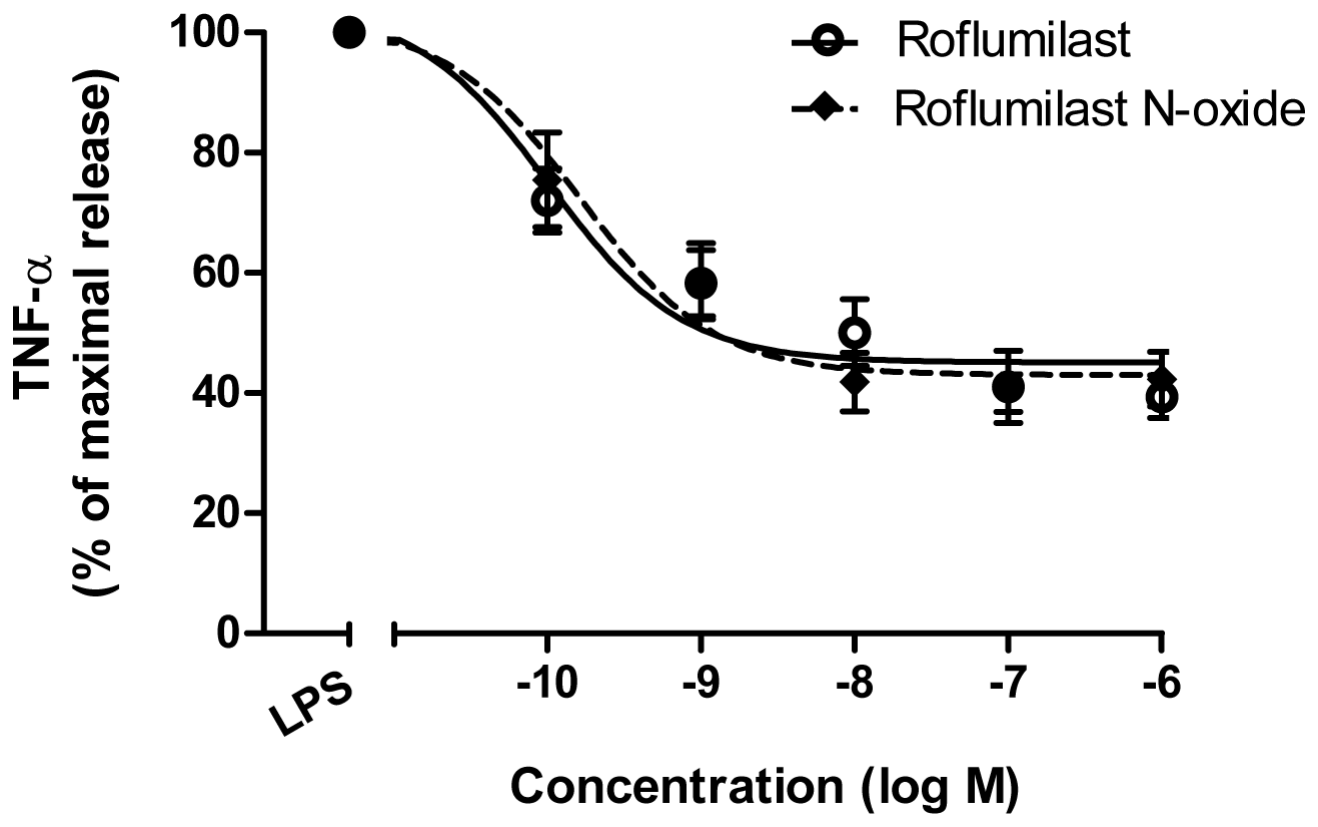
Effects of roflumilast and roflumilast-N-oxide on TNF-α release from LPS-stimulated human lung explants. Lung explants were incubated with roflumilast (solid line) or roflumilast-N-oxide (dashed line) (0.1-1000 nM) or vehicle prior to addition of LPS (1 µg/mL) and incubated for a further 24h. Values are the means ± S.E.M. of seven experiments.

Lipopolysaccharide induced an approximately 100-fold increase in TNF-α release (from 59 ± 11 pg/100 mg in the absence of LPS to 6170 ± 788 pg/100 mg in the presence of LPS; n = 7). Roflumilast and roflumilast-N-oxide reduced this increase in a concentration-dependent manner and with similar potency, with pD_2_ values of 9.9 ± 0.2 (corresponding to an EC_50_ of 0.12 nM) and 9.7 ± 0.2 (corresponding to an EC_50_ of 0.2 nM) (n = 7), respectively. Regardless of the high potency, efficacy was relatively low; each compound did not inhibit more than around 55% of the LPS-induced TNF-α release (Figure 3). Roflumilast did not alter the very weak TNF-α release from non-stimulated lung explants (with a median [range] of 44 [27-72] pg/100 mg in the absence of roflumilast and 39 [22-61] pg/100 mg in the presence of roflumilast, n = 3).

### Effects of roflumilast and roflumilast-N-oxide on chemokine release from LPS-stimulated human parenchymal explants

Non-stimulated lung parenchymal explants produced variable amounts of chemokines, with CCL2, CXCL1 and CXCL8 showing the highest baseline levels of release (Table 2). Addition of LPS (1 µg/mL) resulted in variable increases in chemokine release. The largest increases were observed for CCL3 (x20), CCL4 (x17) and CXCL10 (x77) (Table 1). In contrast, addition of LPS increased the release of the CXCR2 ligands CXCL1, CXCL5 and CXCL8 by a factor of just 3 to 4. Complete and selective inhibition of PDE4 (with 100 nM roflumilast or roflumilast-N-oxide, respectively) partially reduced the LPS-induced release of CCL2, 3 and 4, CXCL9 and CXCL10. However, CXCL1, CXCL5 and CXCL8 release was unaffected (Table 2). The chemokines’ degrees of responsiveness to PDE4 inhibition by 100 nM roflumilast ranged from an 83% reduction for CXCL10 to a 42% reduction for CCL2 (Table 1). We found that roflumilast-N-oxide at 1 nM (a concentration that corresponds to therapeutic plasma levels [1,6,7]) significantly reduced the release of CCL2, 3, 4, CXCL9 and CXCL10 (ranging from a 23% reduction for CCL2 to a 44% reduction for CXCL10). The extent of inhibition was concentration-dependent for both roflumilast and roflumilast-N-oxide. The two PDE4 inhibitors showed comparable effects on the release of each of the investigated chemokines. Neither roflumilast nor roflumilast-N-oxide altered the weak release of any of the investigated chemokines from non-stimulated lung explants (n = 3).

**Table 2:** The effect of roflumilast and roflumilast-N-oxide on LPS-induced chemokine release in culture supernatants of human lung explants.

Chemokine (n)	Baseline	LPS only	LPS + R (1 nM)	LPS + R (100 nM)	LPS + RNO (1nM)	LPS + RNO (100 nM)
CCL2 (n = 7)	43.3 ± 11.0	262.5 ± 43.7	203.2 ± 37.4* (24%)	143.3 ± 24.3*** (42%)	204.1 ±36.4* (23%)	161.9 ± 32.1*** (36%)
CCL3 (n = 6)	1.5 ± 0.7	30.0 ± 7.4	17.3 ± 5.1** (45%)	10.2 ± 2.5*** (65%)	18.4 ± 4.1** (38%)	10.1 ± 3.9*** (70%)
CCL4 (n = 7)	2.2 ± 1.0	37.9 ± 4.8	26.1 ± 4.2*** (33%)	14.7 ± 2.9*** (62%)	23.2 ± 2.9*** (38%)	17.0 ± 3.2*** (57%)
CXCL1 (n = 7)	25.4 ± 6.3	98.5 ± 33.5	105.4 ± 30.0	103.6 ± 33.3	109.2 ± 37.2	116.7 ± 37.9
CXCL5 (n = 5)	7.5 ± 1.3	28.4 ± 3.1	34.3 ± 2.3	32.5 ± 3.4	30.2 ± 6.8	32.9 ± 3.3
CXCL8 (n = 5)	172.9 ± 23.7	575.0 ± 57.8	643.9 ± 95.0	527.9 ± 24.9	570.2 ± 92.4	692.1 ± 75.1
CXCL9 (n = 6)	4.4 ± 2.7	40.2 ± 10.9	23.0 ± 6.8* (40%)	10.2 ± 2.6*** (69%)	22.6 ± 2.6* (41%)	11.4 ± 2.1*** (67%)
CXCL10 (n = 5)	0.04 ± 0.01	3.1 ± 0.9	1.5 ± 0.7* (57%)	0.6 ± 0.3*** (83%)	1.5 ± 0.6* (44%)	0.7 ± 0.2*** (70%)

Lung explants were incubated with medium (baseline) or LPS (1 µg/mL) in the absence or presence of roflumilast (R) at 1 nM or 100 nM or roflumilast-N-oxide (RNO) at 1 nM or 100 nM. Supernatants were harvested at 24h and analyzed for cytokine levels with an ELISA. Results are expressed in ng/100 mg of lung tissue. Values are means ± S.E.M. for between five and seven preparations from different patients. The percentage inhibition produced by the PDE4 inhibitors (calculated for each experiment separately) is given in brackets. *p<0.05; **p<0.01; ***p<0.001 vs. control experiments (LPS only).

**Figure 4:**
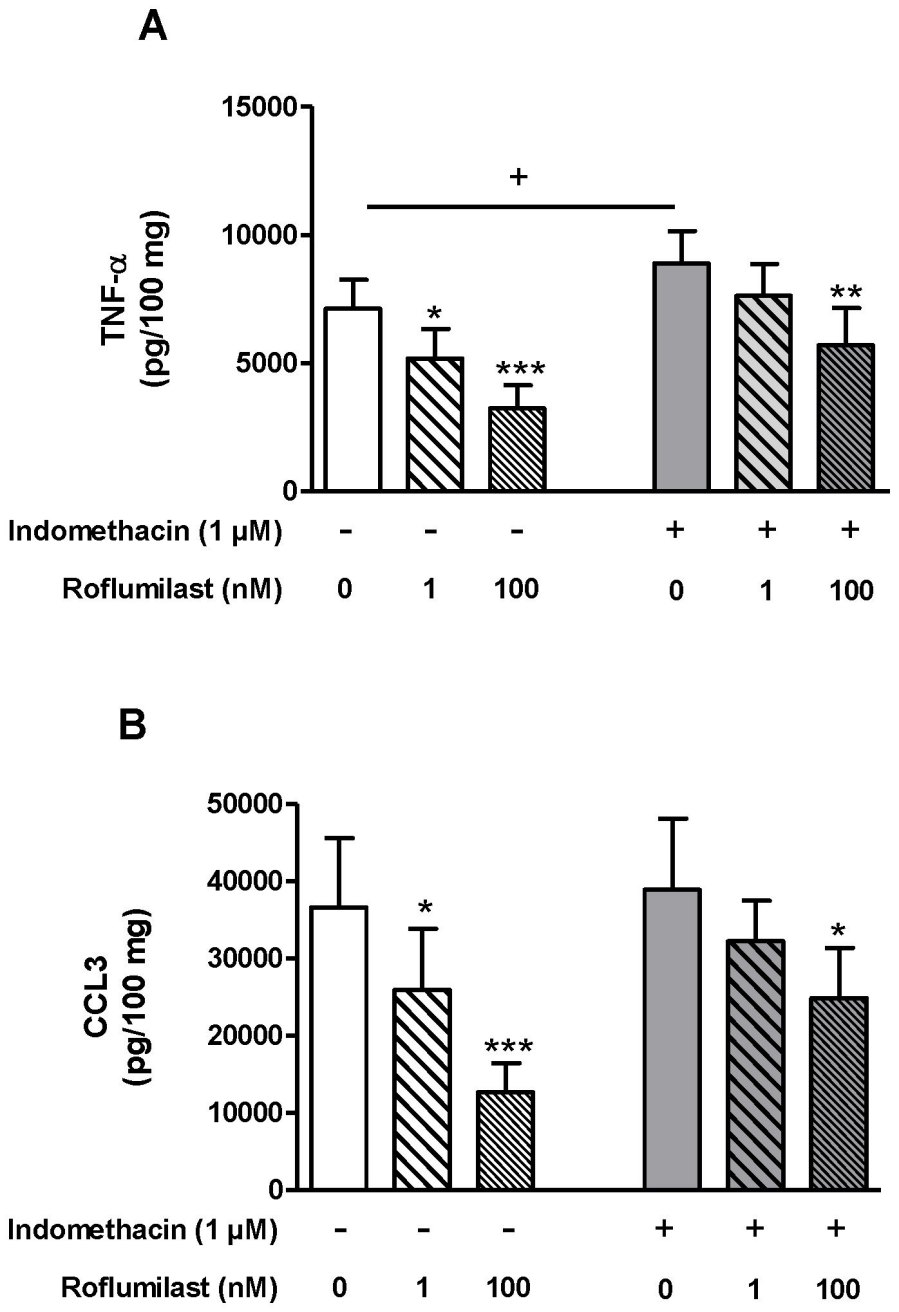
Effects of indomethacin on TNF-α (A) and CCL3 (B) release from LPS-stimulated human lung explants. Lung explants were pre-incubated with indomethacin (1 µM) in the presence or absence of roflumilast (1 nM and 100 nM) or with vehicle prior to LPS (1 µg/mL) challenge for 24h. Values are the means ± S.E.M. for seven different donors. ^*^ *p*< 0.05; ^**^ *p*< 0.01; ^***^ *p*< 0.001 vs. its respective control. ^+^ *p*< 0.05 vs. the LPS-free control.

Previous studies in human monocyte-derived macrophages have shown that the addition of PGE_2_ (in order to increase cAMP formation) was required to observe a reduction in LPS-stimulated TNF-α release by PDE4 inhibitors [5]. In human pulmonary macrophages, fibroblasts or chondrocytes, autocrine prostaglandins (and PGE_2_, in particular) enabled PDE4 inhibitors to exert anti-inflammatory effects [1,5,24]. In the present study of LPS-stimulated human lung explants, roflumilast alone was able to curb the release of TNF-α and chemokines (as detailed above). In order to characterize the putative role of endogenously produced prostanoids in transducing PDE4 inhibition into a reduction in mediator release, we explored the effect of the non-selective cyclooxygenase inhibitor indomethacin. Indomethacin markedly countered roflumilast’s inhibitory effects on the release of cytokines (as exemplified by TNF-α and CCL3) (Figure 4). Furthermore, substantial amounts of PGE_2_ accumulated in culture supernatants from explants over 24h in the presence or absence of LPS (2349 ± 475 pg/mL and 2042 ± 820 pg/mL (n = 7), respectively). Concurrent incubation with indomethacin abolished the accumulation of PGE_2_ (with concentrations below the LOD (n = 7)). Roflumilast did not alter PGE_2_ production (2776 ± 919 pg/mL (n = 7)). Taken as a whole, these results suggest that the endogenous synthesis of prostanoids (such as PGE_2_) acts together with the PDE4 inhibitor to reduce the LPS-stimulated release of cytokines from lung explants.

### Effects of roflumilast, formoterol and forskolin on LPS-induced TNF-α and chemokine release

**Figure 5:**
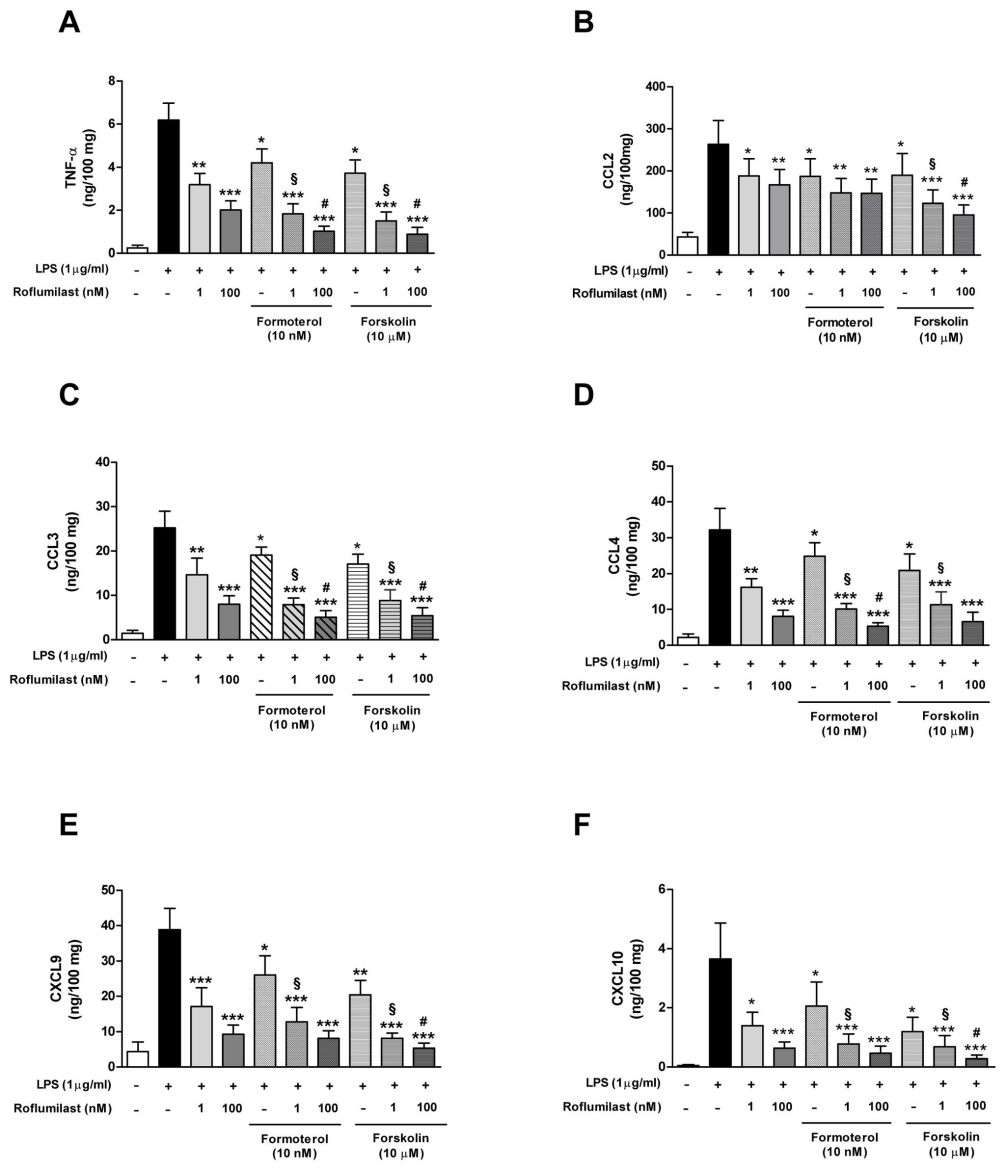
Effects of formoterol or forskolin (alone and combined with roflumilast) on LPS-induced TNF-α and chemokine release. The explants were incubated with roflumilast (1 and 100 nM), formoterol (10 nM), forskolin (10 µM) or vehicle prior to LPS (1 µg/mL) stimulation for 24h. TNF-α (A), CCL2 (B), CCL3 (C), CCL4 (D), CXCL9 (E) and CXCL10 (F) were then measured in culture supernatants. Values are the means ± S.E.M. of eight experiments. ^*^ *p*<0.05; ^**^ *p*<0.01; ^***^ *p*<0.001 vs. LPS; **^§^** *p*<0.05 vs. 1 nM roflumilast; **^#^** *p*<0.05 vs. 100 nM roflumilast.

In this series of experiments, the magnitudes of LPS-induced increase in TNF-α and chemokine release (Figure 5, Table 3) were similar to those described above (Table 2). In order to explore the effects of exogenous cAMP generators on LPS-induced TNF-α and chemokine release in the absence or presence of roflumilast, lung parenchymal explants were incubated with formoterol or the adenylyl cyclase activator forskolin (10 µM) for 30 min before LPS stimulation, in the presence or absence of roflumilast (1 or 100 nM). Formoterol at 10 nM (a concentration considered as optimal in human lung fibroblasts and isolated bronchi [25,26]) decreased the release of (i) CCL3 and CCL4 by about 20-25%, (ii) TNF-α, CCL2 and CXCL9 by about 30-33% and (iii) CXCL10 by about 40% (Figure 5). Concomitant incubation with formoterol and roflumilast (1 nM) produced greater inhibition (relative to roflumilast alone) of the release of TNF-α, CCL3, CCL4, CXCL9 and CXCL10 (Figure 5). For example, the LPS-induced release of CCL3 and CCL4 was respectively reduced by 42% and 50% in the presence of 1 nM roflumilast alone, by 24% and 23% in the presence of 10 nM formoterol alone and by 69% and 68% when both compounds were present. Roflumilast-N-oxide was not tested in this series of experiments because it is known to have essentially the same potency and efficacy as the parent compound.

**Table 3:** The effect of roflumilast on LPS-induced chemokine release in culture supernatants of human lung explants.

	Baseline	LPS only	LPS + R(1 nM)	LPS + R (100 nM)	LPS + For (10 nM)	LPS + R (1 nM) + For	LPS + R (100 nM) + For	LPS + Fsk (10 µM)	LPS + R (1 nM) + Fsk	LPS + R (100 nM) + Fsk
CXCL1	37 ± 6	196 ± 12	179 ± 10	166 ± 8	183 ± 7	167 ± 8	178 ± 11	186 ± 14	157 ± 7	164 ± 7
CXCL5	5 ± 0.5	30 ± 3	29 ± 2	27 ± 1	28 ± 1	32 ± 1	26 ± 2	29 ± 2	30 ± 1	30 ± 1
CXCL8	69 ± 8	520 ± 21	487 ± 24	516 ± 39	567 ± 38	475 ± 35	538 ± 50	473 ± 36	438 ± 31	410 ± 35

Lung explants were incubated with medium (baseline) or LPS (1 µg/mL) in the absence or presence of roflumilast (R) alone at 1 nM or 100 nM or combined with either formoterol (For 10 nM) or forskolin (Fsk 10 µM). Supernatants were harvested at 24h and analyzed for cytokine levels with an ELISA. Results are expressed in ng/100 mg of lung tissue. Values are means ± S.E.M. for eight preparations from different patients. No significant inhibition was observed in control experiments (LPS only).

Forskolin reduced the release of (i) CCL2, CCL3 and CCL4 by approximately 30-35%, (ii) TNF-α and CXCL9 by approximately 40-45% and (iii) CXCL10 by approximately 65% (Figure 5). The roflumilast-forskolin combination produced greater inhibition than roflumilast alone. In both the absence and presence of roflumilast, neither formoterol nor forskolin altered LPS-induced release of CXCL1, CXCL5 and CXCL8 (Table 3). On the whole, there were no differences between explants from ex-smokers and those from smokers in terms of LPS-induced cytokine release or the inhibitory effect of roflumilast.

### Effects of roflumilast and formoterol on cAMP accumulation

**Figure 6:**
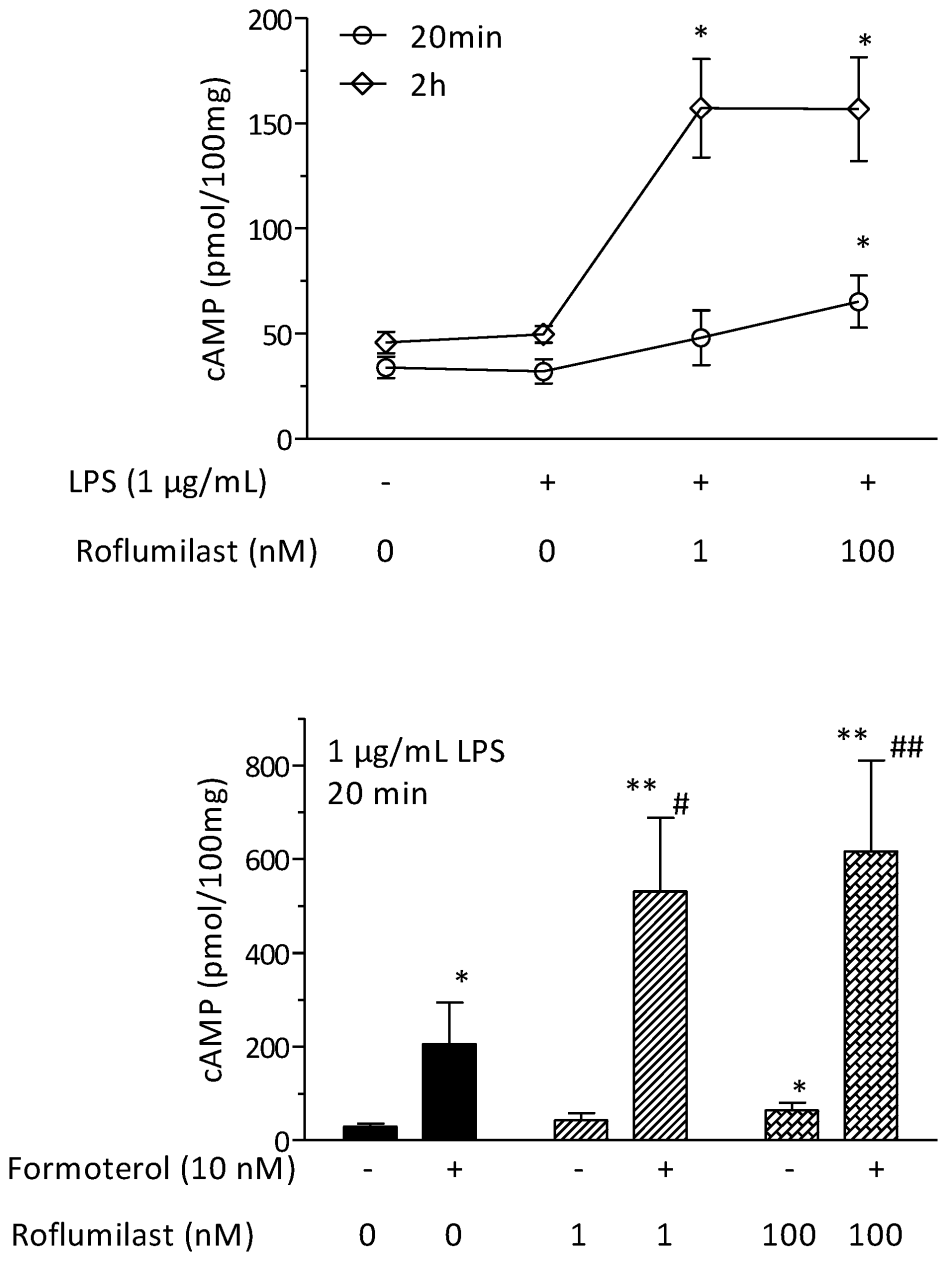
Effects of roflumilast on intracellular cAMP levels. (A) Lung parenchymal explants were incubated for either 20 min or 2h with media and vehicle or with LPS (1 µg/mL) and vehicle or roflumilast at 1 nM or 100 nM. (B) Lung parenchymal explants were exposed to vehicle or roflumilast at 1 nM or 100 nM in the presence or absence of formoterol (10 nM) and with LPS (1 µg/ml) for 20 min. Tissue homogenates were then analyzed for cAMP by EIA. The data shown are the means ± S.E.M. for samples from four different donors. ^*^ *p*< 0.05 vs. vehicle control (A); ^*^ *p*< 0.05; ^**^ *p*< 0.01 vs. LPS, **^#^** *p*<0.05; **^# #^** *p*<0.01 vs. formoterol and LPS (B).

Incubation of human lung explants with LPS for 20 min did not significantly change the cAMP content. In the additional presence of 100 nM roflumilast, the cAMP concentration content rose two-fold, whereas the modest cAMP increase with 1 nM roflumilast was not statistically significant (Figure 6A).

Following a 2h incubation of human lung explants with 1 nM roflumilast and LPS, the cAMP content in the explants increased by 3.5-fold, when compared with vehicle controls. Incubation with 100 nM roflumilast did not yield a further enhancement (Figure 6A).

In another series of experiments, the additional effects of formoterol were explored. Alone, formoterol (10 nM) enhanced the cAMP content in lung explants about 7-fold during a 20-min incubation (in the presence of LPS) (Figure 6B). This effect was amplified by roflumilast at 1 nM or 100 nM (Figure 6B).

## Discussion and Conclusions

Our present results demonstrated that the PDE4 inhibitor roflumilast and its active metabolite roflumilast-N-oxide partially reduced the LPS-induced release of TNF-α and that of the chemokines CCL2, CCL3, CCL4, CXCL9 and CXCL10 (but not the release of the CXCR2 ligands CXCL1, CXCL5 and CXCL8) from human lung explants. The β_2_-adrenoceptor agonist formoterol and the adenylyl cyclase activator forskolin decreased the release of the same array of cytokines from LPS-exposed explants. The addition of formoterol (or forskolin) to roflumilast enhances the latter’s inhibition of cytokine production.

We took advantage of the opportunity to study lung explants that contain the full complement of lung cells in their normal ratios and spatial configuration; as such, they approximated well the *in vivo* conditions in the lung [12-14]. This model enabled us to explore the anti-inflammatory properties of roflumilast and its active metabolite under conditions simulating those seen in the intact lung but in which the effect of the circulation is absent. The kinetics of cytokine release (TNF-α, IL-6, CXCL8, IL-10) in response to exposure of human lung explants to LPS has been described previously [13]. Hackett et al. reported that the pattern of cytokines and chemokines released from LPS-stimulated human lung explants reflects (in part) the profile previously reported in bronchoalveolar lavage (BAL) or induced sputum from patients with COPD and bacterial infections [13]. The present study expands the previous observations to a range of chemokines (CCL2, CCL3, CCL4, CXCL1, CXCL5, CXCL9 and CXCL10) involved in the pathophysiology of COPD and its exacerbations [19]. The endotoxin concentrations used in this study were clinically relevant. In BAL samples from patients with pneumonia or acute respiratory distress syndrome [27-29], the final endotoxin concentrations in the airway surface liquid were estimated to be as high as 0.1–10 µg/mL (after taking account of the 100-fold dilution due to lavage [30]).

Our results in lung explants do not provide evidence on the precise source of cytokines in response to LPS and on the contribution of different lung cells to the inhibitory effect of roflumilast. PDE4 enzymes are expressed in all cell types in the lungs (with the exception of platelets), which necessarily includes the main inflammatory cells (neutrophils, macrophages and CD8+ T-cells) and structural cells (bronchial epithelial cells, fibroblasts, airway smooth muscle cells and microvascular endothelial cells) involved in COPD [1,31]. The almost exclusive expression of PDE4 has been described in neutrophils, whereas other PDE isoenzymes (such as PDE3 or PDE1) contribute to the control of intracellular cAMP levels in macrophages, T-cells and structural cells. In line with literature reports [32], our present results indicate that PDE4 accounts for about 38% of the total cAMP hydrolyzing activity in extracts of human lung explants. We found that PDE3 contributed another 27%. This co-expression of PDEs (other than PDE4) involved in cAMP degradation may account for the fact that only a partial reduction in LPS-induced chemokine and TNF-α release from human lung explants was observed when PDE4 was blocked with 100 nM roflumilast. Importantly, roflumilast-N-oxide at 1 nM (a concentration that corresponds to the range of free plasma levels following repeated oral administration of roflumilast in humans (500 µg.d^-1^)) reduced the LPS-induced release of TNF-α, CCL2, CCL3, CCL4, CXCL9 and CXCL10 by between 23% and 44%. In contrast to our recent report on pulmonary macrophages, we did not observe an increase in PDE4 activity in the presence of LPS. This difference may have been due to the parenchyma explants’ shorter duration of exposure to LPS (4 h, instead of 24 h for the macrophages).

PDE4 inhibitors act by increasing cAMP. As a corollary, most of the downstream effects of these drugs are exerted through activation of the cAMP acceptors protein kinase A or Epac, which inhibit key signaling cascades involved in inflammation or remodeling [33,34]. Indeed, the expression of TNF-α, CCL2, CCL3, CCL4, CXCL9 and CXL10 was previously reported to be curbed by cAMP-dependent pathways in monocytes, macrophages and airway smooth muscle cells [35-38]. In COPD, these chemokines are involved in the enhancement of lung infiltration by CD4+ and CD8+ T-cells, B-cells and macrophages [19,39]. In the context of the present study, it is noteworthy that roflumilast reduced lung infiltration by CD4+ and CD8+ T-cells, B-cells and macrophages in mice exposed to tobacco smoke for 6 months [40]. Furthermore, the PDE4 inhibitor cilomilast reportedly reduces counts of bronchial biopsy CD4+ and CD8+ T cells and macrophages in COPD patients [41].

While roflumilast and roflumilast N-oxide reduced the release of CCL2-4, CXCL9, CXCL10 and TNF-α from LPS-exposed lung explants, the PDE4 inhibitors did not affect the neutrophil chemoattractants CXCL1, CXCL5 and CXCL8. Accordingly, it is known that the release of CXCL1 and CXCL8 (in human monocytes/macrophages or airway smooth muscle cells) or CXCL8 (in human bronchial epithelial cells or lung fibroblasts) is barely or not at all affected by compounds that increase cAMP levels (e.g. β_2_-adrenoceptor agonists, forskolin and PDE4 inhibitors) [8,42-45]. In contrast, roflumilast reduces LPS- or tobacco smoke-induced BAL neutrophil influx in mice and rats [1], BAL neutrophil accumulation following bronchial LPS challenge in human volunteers [46] and sputum neutrophil counts in COPD patients [47]. Given that roflumilast inhibits the adhesion of neutrophils to endothelial cells *in vitro* and attenuates LPS-induced leukocyte-endothelial interactions in rats *in vivo* [48], roflumilast likely alleviates neutrophil airway infiltration through its ability to directly limit neutrophil activation.

At a concentration of 10 nM, formoterol partly reduced the LPS-induced release of TNF-α, CCL2, CCL3, CCL4, CXCL9 and CXCL10. This concentration of formoterol has been shown to suppress the expression of cytokines in human airway epithelial cells [49,50], smooth muscle cells [44], monocytes derived-macrophages [42] and human lung fibroblasts [25]. Addition of formoterol to the PDE4 inhibitor (1 nM roflumilast) increased the inhibitory effects on the LPS-induced release of chemokines and TNF-α. In mechanistic terms, one can expect a PDE4 inhibitor and a β_2_-adrenoceptor agonist to show synergistic effects when present together. This type of synergistic effect has been reported in human eosinophils and neutrophils; however, PDE4 accounts for virtually all the cAMP metabolizing capacity in these cell types [51,52]. Only additive effects have been observed with either β_2_-adrenoceptor agonist or other stimulants of adenylyl cyclase in human peripheral blood mononuclear cells, blood monocytes or human pulmonary macrophages (which express other cAMP-hydrolyzing PDEs) [8,53,54]. In the present study of lung explants, the objective was to imitate the *in vivo* environment as closely as possible, with mutually interacting cells expressing different cAMP-hydrolyzing PDEs and a degree of PDE4 inhibition in the clinically effective and tolerable range. In such a complex system, a synergistic effect of either a β_2_-adrenoceptor agonist or an adenylyl cyclase activator (forskolin) and a PDE4 inhibitor was unlikely to occur.

The non-selective cyclooxygenase inhibitor indomethacin inhibits the effects of roflumilast, as has already been described in human lung fibroblasts and pulmonary macrophages [8,55]. This observation suggests that endogenous prostanoids capable of inducing cAMP synthesis (such as PGE_2_) are produced by the cells in the lung explants and thus supports the hypothesis in which PDE4 inhibitors reduce the release of cytokines. The *in vitro* production of prostanoids by lung parenchyma has been reported previously [56] and *in vitro* production of PGE_2_ was observed in the present study. The lack of synergy between formoterol and roflumilast may also be due to the release of endogenous prostanoids by cells within the lung explants, since these prostanoids also interfere with cAMP synthesis. In a clinical setting of COPD, concomitant use of a LABA and roflumilast may help to extend the time to first exacerbation [57].

The roflumilast (1 nM)-induced decrease in the release of chemokines and TNF-α from the explants was paralleled by an increase in the explant’s cAMP content after a 2h incubation in the presence of LPS. However, there was almost no increase in the first 20 min of exposure. It could be that during the longer (2h) incubation period, endogenous mediators favoring cAMP generation co-operate with 1 nM roflumilast and raise cAMP levels. In studies of guinea pig macrophages and the human monocyte cell line U937, the PDE4 inhibitor rolipram had little effect on cAMP accumulation [58,59]; cAMP accumulation was only observed in the presence of β_2_-adrenoceptor agonists (resulting in synergistic effects). In the present study, roflumilast and formoterol showed more than additive effects on cAMP levels but not on cytokine release. This disparity may be due to differences in (i) the cell types functionally involved in chemokine and TNF-α production vs. those governing the cAMP response and (ii) the complex intracellular pathways linking cAMP and cytokine production.

Our investigations on the effect of roflumilast were conducted on tissue of patients with normal lung function. Roflumilast is indicated for the treatment of COPD patients and it has been suggested that parenchyma tissue of patients with mild (GOLD I) or moderate (GOLD II) COPD would respond with an enhanced inflammatory response following exposure to LPS [13]. Future studies would be required to determine if the anti-inflammatory effect of roflumilast is altered in lung explants from COPD patients. In summary, the PDE4 inhibitors roflumilast and roflumilast-N-oxide reduced the LPS-induced release of TNF-α from human lung parenchymal explants to a significant extent and the release of the chemokines CCL2, CCL3, CCL4, CXCL9 and CXCL10 to a moderate extent. Although the β_2_-adrenoceptor agonist formoterol increased the anti-inflammatory effect of roflumilast in this experimental system, the combination was not synergistic.
